# Scientific Items

**Published:** 1885-08-20

**Authors:** 


### SCIENTIFIC ITEMS.

Soleina is the name given to a new burning fluid recently invented by Professor Guillemar, of Paris, which is thus described in Il Progresso: The liquid gives a light as vivid almost as that of the sun—so intense, indeed, that it cannot be looked at without protecting the eyes. At first it was thought to be a derivative of petroleum, but in fact is something entirely different, being the result of distillation of the resin of the pine and other balsamic trees, and thus closely connected with oil of turpentine. It possesses many advantages over petroleum; a pleasant odor, resembling that of incense; its eminations are not unhealthy; its light is white, and has no shade or tinge of red or yellow. This last fact makes the new light of inestimable advantage to painters, artists, dressmakers, and others who have to work in or distinguish colors by artificial light. The great drawback to the rapid introduction of the fluid is the fact that it cannot be burned in the ordinary petroleum or fluid lamps, but requires a burner and vessel of peculiar construction, which is also the invention of Professor Guillemar.—*National Druggist.*

**Our National Banking System.**—The ever-recurring question as to the methods which should be adopted for supplying the country with currency promises soon again to demand attention, and to be beset with all its old-time perplexities. It is the riddle which is presented in turn to each civilized nation, and, although the penalty of default is severe, no satisfactory answer has as yet been found.

The national banking system, which has frequently been declared to be the best yet devised, cannot be said to offer a solution, for, although it served its temporary purpose very well, it lacks, so far as its currency is concerned, the essential element of permanency, being based upon a public debt that, fortunately, is not a perpetuity. Recently great concern has been felt and expressed over the prospective contraction, if not total withdrawal, of the national bank circulation which is likely to result from the diminished supply of Government bonds. The prospect is generally deplored. Sundry bills were introduced into the late Congress, looking to a mitigation or postponement of the consequent evils, but no conclusive action was taken, nor is there much reason to expect that the subject will receive serious congressional consideration until it compels attention.—From the "Future of National Banking," by E. R. Leland, in *Popular Science Monthly for Aug.*

**The Pain of being Hung.**—Dr. Taylor states that "death from hanging appears to take place very rapidly, and without causing any suffering to the person. Professor Tidy, also, speaks of the painless nature of death from hanging; while Professor Haughton, in his paper read before the Surgical Society of Dublin, says that "the old system of taking a convict's life by suffocation is inhumanly painful, unnecessarily prolonged, and revolting to those whose duty it is to be present." Those who speak of the painless nature of death by strangulation arrive at this conclusion from the fact that many cases of suicide are not completely suspended, and that if they wished they could easily relieve the constriction by assuming the erect posture, and in other cases of recovery from attempted suicide by hanging there is no recollection of any suffering. It should be remembered, however, that there is a great difference between the mental attitude of the suicide and one who is about to suffer the extreme penalty of the law. In the former case he is regardless, and perhaps also not very sensitive, of a little suffering, while in the latter every nerve is braced up to resist the inevitable result. Moreover, in those cases of recovery the loss of recollection of suffering does not prove that there was none. It might as well be said that because in many cases of recovery from meningitis there was no remembrance of any suffering, therefore there was none. No doubt, the pain in hanging can under no circumstances be very acute, yet when we see a culprit heaving his chest and almost raising the whole body in his struggles for breath we must conclude that there is at least a considerable amount of mental torture.—Dr. James Baar, in *Popular Science Monthly for August.*

**Hindoo Time.**—The Hindoos also employed ages in the computation of time, and these, too, divided into two periods of different durations. The present age is the *kali yuga*, or the age of iron; 4,985 years of it have already passed, but its total duration is supposed to be 432,000 years. The succession of the ages, counting back, is given as follows:

Fourth age—*Kali yuga*, age of iron, or of woe (the present age), to be of 432,000 years.

Third age—*Dvapara yuga*, 864,000 years.

Second age—*Treta yuga*, or age of silver, 1,296,000 years.

First age—*Krita yuga*, age of gold, or of innocence, 1,728,000.

These four ages form the *maha yuga*, or great age, of 4,320,000 years. The length of a patriarchate is seventy-one maha yugas, or 306,720,000 years, to which is added a twilight period of 1,728,000 years, making in all 308,448,000 years. Fourteen of these patriarchates, augmented by a dawn of 1,728,000 years, gives 4,320,000,000 years, which form a *kalpa*, or the *æon* of the Hindoo chronology.

A kalpa is only a day in the life of Brahma, whose nights are also of the same duration. Now, Brahma lives a hundred years of three hundred and sixty days and three hundred and sixty nights. The present epoch is the *kali yuga* of the twenty-seventh grand age of the seventh patriarchate of the first æon of the second half of the life of Brahma, who is now in his 155,521,972,848,985th spring. Yet the whole life of Brahma is only a little longer tan a single wink of Sivia's eye!—From "Curiosities of Time-Reckoning," by M. L. Barre, in *Popular Science Monthly for August.*

